# A dopamine-methacrylated hyaluronic acid hydrogel as an effective carrier for stem cells in skin regeneration therapy

**DOI:** 10.1038/s41419-022-05060-9

**Published:** 2022-08-27

**Authors:** Meihua Gong, Fei Yan, Li Yu, Furong Li

**Affiliations:** 1 Department of Plastic and Cosmetic Surgery, Shenzhen People’s Hospital (The First Affiliated Hospital, Southern University of Science and Technology; The Second Clinical Medical College of Jinan University), Shenzhen, 518055 Guangdong China; 2 Translational Medicine Collaborative Innovation Center, Shenzhen People’s Hospital (The First Affiliated Hospital, Southern University of Science and Technology; The Second Clinical Medical College of Jinan University), Shenzhen, 518055 Guangdong China; 3 Guangdong Engineering Technology Research Center of Stem Cell and Cell Therapy, Shenzhen Key Laboratory of Stem Cell Research and Clinical Transformation, Shenzhen Immune Cell Therapy Public Service Platform, Shenzhen, 518020 Guangdong China

**Keywords:** Biomedical materials, Regeneration

## Abstract

Adipose-derived stem cells (ADSCs) show potential in skin regeneration research. A previous study reported the failure of full-thickness skin self-repair in an injury area exceeding 4 cm in diameter. Stem cell therapies have shown promise in accelerating skin regeneration; however, the low survival rate of transplanted cells due to the lack of protection during and after transplantation leads to low efficacy. Hence, effective biomaterials for the delivery and retention of ADSCs are urgently needed for skin regeneration purposes. Here, we covalently crosslinked hyaluronic acid with methacrylic anhydride and then covalently crosslinked the product with dopamine to engineer dopamine-methacrylated hyaluronic acid (DA-MeHA). Our experiments suggested that the DA-MeHA hydrogel firmly adhered to the skin wound defect and promoted cell proliferation in vitro and skin defect regeneration in vivo. Mechanistic analyses revealed that the beneficial effect of the DA-MeHA hydrogel combined with ADSCs on skin defect repair may be closely related to the Notch signaling pathway. The ADSCs from the DA-MeHA hydrogel secrete high levels of growth factors and are thus highly efficacious for promoting skin wound healing. This DA-MeHA hydrogel may be used as an effective potential carrier for stem cells as it enhances the efficacy of ADSCs in skin regeneration.

## Introduction

Although skin has a strong self-repair ability following epidermal injury, this process is hindered when the deep dermal layer becomes injured [1]. A previous study reported the failure of full-thickness skin self-repair in an injury area exceeding 4 cm in diameter [2]. Failure of the physiological repair response leads to either a hypertrophic scar or a chronic refractory wound [1]. The standard therapies for full-thickness skin defects mainly include autologous skin grafting and flap grafting; notably, autologous skin grafting requires a sufficient volume of skin and the availability of donor sites may be limited by extensive skin defects or pathological skin diseases [3]. Clinical studies mainly focus on enhancing the healing of wounds that are difficult to self-repair, maximizing the recovery of biological function and minimizing esthetic impacts remain [1]. The cost of skin wound repair accounts for approximately half of global annual medical expenses [4]. Quality treatment methods for the repair of large-area skin defects remain limited [5], and more effective therapies for wound healing are urgently needed [6].

Stem cell therapies have shown promise for accelerating skin regeneration [7]. Mesenchymal stem cells (MSCs) are being investigated for skin wound healing applications, as they accelerate the formation of granulation tissue and blood vessel ingrowth and reduce the inflammatory response [8]. Adipose-derived stem cells (ADSCs) have some advantages over other MSCs, as they are abundantly obtained from stem cell donors, have an excellent proliferative ability and have multidirectional differentiation potential[9, 10]; moreover, ADSCs are hindered by fewer ethical problems than other types of MSCs [11].

However, the effectiveness of stem cell therapies is low due to poor engraftment and low rates of cell survival after transplantation into harsh environments, such as large skin defects [12]. Compared to cells injected alone, the wound healing potential of stem cells encapsulated in biomaterials may be optimized by stem cell homing to the defect site and increasing the secretion of biologically active factors [8]. Extracellular matrix (ECM) components are actively interconnected to create unique topographies, which crucially provide cells with biochemical and structural support, and the active cell-ECM interaction controls cell behaviors, such as proliferation, differentiation and migration [13, 14]. Cells probe the stiffness of their niche using mechanotransduction to interact with the ECM and surrounding cells, sense mechanical perturbations, and transmit environmental pressure to modulate their behaviors [15]. In addition to functions of cell scaffolding, the ECM serves as a dynamic communicative plane to its circumambient cells in the tissue to maintain tissue integrity [16]. Hydrogels are characterized by unique hydrated networks and physicochemical properties and have been widely used as scaffolds for cell therapies based on their ability to mimic the ECM [13, 17]. Hyaluronic acid (HA) is an ECM component with good biocompatibility and hydrophilicity and is a common natural polymer used for cell scaffolds [18]. However, pure HA has poor mechanical properties, low resistance to enzymatic degradation [19, 20], and a high swelling rate; thus, it is difficult to self-assemble into macromolecules in solution because of its uniform repeating structure [21]. These disadvantages have hindered its use as a wound dressing because it is unable to sustain the structure of bioscaffolds [22]. The combination of biomaterials and stem cells is known to be very practical for further accelerating wound healing [23].

Therefore, the aim of the present study was to develop a new biomaterial for use as a carrier of stem cells based on a biocompatible natural material (HA) and typical cell growth scaffold for skin regeneration therapy. The effect of the modified HA hydrogel on stem cells during skin regeneration was verified by performing in vitro and in vivo analyses.

## Materials and methods

### Ethics statement

All animal experiments were approved by the Institutional Animal Care and Use Committee at Jinan University and performed according to National Institutes of Health guidelines. Seven- to ten-day-old BALB/c mice and 6-week-old female BALB/c mice were obtained from the Experimental Animal Center of Guangdong Province and housed under standard conditions according to the regulations of the Ethics Committee of the Medical Sciences Department. Six-week-old female BALB/c mice were group-housed in a temperature-controlled room (24 ± 1 °C) with 50–60% humidity. The study was performed in accordance with the Declaration of Helsinki. The experiments were approved by the Laboratory Animal Ethics Committee of Jinan University (Guangzhou, Guangdong Province, China, IRB number. 20210228-12).

### Dopamine-methacrylated hyaluronic acid (DA-MeHA) synthesis

First, the methacrylate group was covalently crosslinked to HA to synthesize methacrylated hyaluronic acid (MeHA) [19].

HA (1 g, H823435, Macklin, Shanghai, China) was dissolved to a concentration of 1 w/v% in double deionized water (DDI H_2_O). Methacrylic anhydride (276685-500 ml, Sigma-Aldrich, St. Louis, Missouri, United States) was added dropwise (~2.5 mL) while stirring the solution at 4 °C. The stirring mixture was maintained at pH ~8 by the continuous addition of 4 M NaOH for 8 h, followed by a reaction overnight at 4 °C and subsequent addition of methacrylic anhydride (~1 mL) at pH ~8 for ~4 h to synthesize MeHA. The extra methacrylic anhydride was dialyzed against DDI H_2_O for 3 days, frozen at −80 °C, lyophilized, and stored at −80 °C in a spongy form [19].

In the second step, the dopamine group was covalently crosslinked to MeHA to synthesize DA-MeHA.

MeHA (1 g) was completely dissolved in 100 mL of DDI H_2_O (pH~5.5). Carbodiimide hydrochloride (EDC, 506.0 mg, 03450-25 g, Sigma-Aldrich, St. Louis, Missouri, United States) and N-hydroxysuccinimide (NHS, 303.7 mg, 56480-100 g, Sigma-Aldrich, St. Louis, Missouri, United States) were added to the MeHA solution. After a 30 min incubation, dopamine hydrochloride (500 mg, H8502-25g, Sigma-Aldrich, St. Louis, Missouri, United States) was added to the solution for incubation with gentle stirring at 25 °C for 12 h to synthesize DA-MeHA. Then, the solution was purified by dialysis in acidified DDI H_2_O (pH~5) at 25 °C for 12 h. The dialyzed solution was frozen at −80 °C, lyophilized, and stored at −80 °C until use.

### Characterization of DA-MeHA

DA-MeHA was characterized using ^1^H NMR (400 MHz, JNM-ECX-400P, JEOL, Japan) in D_2_O. The crosslinking degrees of methacrylate and dopamine in DA-MeHA were calculated.

#### Thermogravimetric analysis (TGA)

The thermal degradation behaviors of HA, MeHA, and DA-MeHA were characterized using TGA with a thermal analyzer (PerkinElmer, STA-8000, USA). Approximately 20 mg of HA, MeHA, and DA-MeHA were placed in an alumina crucible for measurement and heated from 30 °C to 650 °C in nitrogen gas at a heating rate of 20 °C/min [24].

#### Scanning electron microscopy (SEM)

HA (powder), MeHA (freeze-dried) and DA-MeHA (freeze-dried) were prepared for observation using SEM. The specimens were coated with gold using a sputtering device prior to the SEM observation. The structural features of the hydrogels were monitored with a scanning electron microscope (Hitachi Regulus 8100, Japan), and micrographs of the samples were recorded.

### The isolation and formation of spheroids composed of ADSCs

ADSCs were isolated as described in our previous study [25]. Briefly, Seven- to ten-day-old BALB/c mice were sacrificed by intraperitoneally injecting an overdose of pentobarbital sodium and soaked in 75% alcohol for 1 h. Then, the subcutaneous fat tissues were harvested quickly, rinsed with phosphate‐buffered saline (4 °C) and digested with 0.1% collagenase I (17100-017, Invitrogen, California, United States) with gentle shaking (100 rpm) at 37 °C for 45–55 min. Then, the sample was centrifuged at 1200 rpm for 10 min, and the upper suspended tissue was discarded. The isolated ADSCs were cultured in low-glucose DMEM containing 10% fetal bovine serum and 1% antibiotics/antimycotics at 37 °C with 5% CO_2_.

The third or fourth generation of ADSCs (~2.5–5 × 10^4^ cells/well) were aggregated into round-bottom ultralow attachment 96-well plates for 48 h to form spheroids composed of ADSCs.

The viability and proliferation of the ADSCs in spheroids were assessed as follows. First, the ADSCs were stained with Cell tracker^TM^ CM-Dil (C7000, Invitrogen, California, United States) and then cultured in round-bottom ultralow attachment 96-well plates for 48 h. The formed spheroids composed of ADSCs were plated in a 6-well plate for 2 days, fixed with a solution of 4% paraformaldehyde (P6148-1KG, sigma, St. Louis, Missouri, United States) and observed under a confocal microscope (Leica TCS SP8, Germany).

### In vitro assay of DA-MeHA biocompatibility

For DA-MeHA hydrogel formation, DA-MeHA was dissolved at 1.0 w/v% and 1.5 w/v% in phosphate-buffered saline (pH 7.4) with a photoinitiator (0.2 w/v% lithium phenyl (2,4,6-trimethylbenzoyl) phosphinate, LAP; L157759, Aladdin, Shanghai, China) and stirred at 25 °C for 24 h. The 2.0 w/v% DA-MeHA hydrogel was too viscous and difficult to stir evenly. Sterilization of the DA-MeHA hydrogel was carried out at 70 °C for 30 min and 4 °C for 10 min for a total of 3 cycles.

We conducted live/dead viability and cell counting kit-8 experiments to assess the effects of the DA-MeHA hydrogel on cell adhesion and proliferation.

#### Live/dead viability assay

ADSCs were trypsinized and seeded at a density of ~30,000 cells/well in normal growth media on 12-well plates covered with different concentrations of the DA-MeHA hydrogel: blank (named the control group), 1 w/v% DA-MeHA hydrogel (named the 1.0 w/v% DA-MeHA group) and 1.5 w/v% DA-MeHA hydrogel (named the 1.5 w/v% DA-MeHA group). At the desired time points (1, 3, 5, and 7 d), the normal medium was removed, and the plates were washed with PBS three times. The prepared live/dead assay reagents calcein AM and propidium iodide (KGAF001, KeyGEN BioTECH, Shanghai, China) were added to the plates for 10 min of incubation at 37 °C. Then, the samples were washed with PBS twice and imaged using a confocal microscope (Leica TCS SP8, Germany).

#### Cell counting kit-8 (CCK-8) assay

Cells were seeded on 24-well plates at a density of ~10,000 cells/well with normal growth media and then divided into the control group, 1 w/v% DA-MeHA hydrogel group and 1.5 w/v% DA-MeHA hydrogel group. CCK-8 (KGA317s-500, KeyGen BioTECH, China) dye solution (300 μL of DMEM with 30 μL of CCK-8 reagent) was added to each well on days 1, 2, 3, 4, 5, 6, and 7. Wells containing only DMEM and no cells were used as the blank control. After an incubation at 37 °C for 40–90 min, the supernatant was transferred onto a 96-well plate, and the absorbance was detected at 450 nm using a spectrophotometer (Multiskan GO, Thermo Scientific, United States). The results were calculated using the formula OD_t_/OD_0_ × 100% (OD, optical density).

### In vivo experiment

The therapeutic effect of the DA-MeHA hydrogels encapsulating ADSCs on skin regeneration was evaluated using a full-thickness skin defect model. Thirty-two 6-week-old female BALB/c mice (20 ± 2 g) were randomly assigned to the control group (untreated), skin defect group, hydrogel group, or hydrogel+ADSCs spheroids group (*n* = 8 mice per group). The animals in each group were treated in sequential order. First, the mice in the treated groups were anesthetized with an intraperitoneal injection of pentobarbital sodium, and the dorsal skin was shaved and sterilized with 0.5% iodophor. Then, a circular full-thickness skin defect with a diameter of 2 cm was created on the backs of the mice (excluding the control group), comprising the dermis and epidermis [3, 5, 26]. Second, the 1.5 w/v% DA-MeHA hydrogels were gently mixed with the spheroids composed of ADSCs using two syringes connected with a sterile connector. The skin wounds were dressed with 500 µl of 1.5 w/v% DA-MeHA hydrogel with 0.2 w/v% LAP and a small amount of potassium sodium periodate solution mixed with or without the spheroids composed of ADSCs (containing ~8 × 10^6^ cells per wound). Third, the DA-MeHA hydrogels with or without ADSCs were activated to cure the skin wound in situ by illuminating them with blue light at a wavelength of 405 nm, and the curing time was less than 30 s. Wounds were covered with a transparent semiocclusive dressing (Tegaderm, 3 M, United States) to ensure that the hydrogels remained stable in the wound and to prevent skin shrinkage at the edge of the wound.

Another in vivo experiment was conducted to further assess the effects of the DA-MeHA hydrogel on the therapeutic efficacy of ADSCs in skin regeneration. Forty 6-week-old female BALB/c mice (20 ± 2 g) were randomly assigned to the control group, skin defect group, ADSCs group, hydrogel group and hydrogel+ADSCs group (*n* = 8 mice per group, containing a suspension of ~8 × 10^6^ ADSCs per wound in the ADSCs group and hydrogel+ADSCs group). Photographs were captured on the 7th and 14th days after surgery.

The wound closure analysis included mainly skin re-epithelialization, hair growth in areas of skin regeneration, and wound closure rate.

#### Wound closure measurement

The wound area was measured using ImageJ software. At the defined time points (days 7 and 14), the wound area was measured, and the wound healing rate was defined using the following formula:

$${{{\mathrm{Wound}}}}\;{{{\mathrm{closure}}}}\;{{{\mathrm{rate}}}} = 1 - \frac{{A_t}}{{A_0}} \times 100\%$$

where A_0_ is the original wound area and A_t_ is the area of wound in the photos captured at the respective time points.

On the 14^th^ day after surgery, the mice (*n* = 3 per group) were anesthetized with an intraperitoneal injection of pentobarbital sodium and perfused with saline through the ascending aorta, followed by 4% paraformaldehyde in 0.1 M phosphate buffer [27]. Skin samples from the original skin wound defects were obtained and divided into two equivalent pieces used for hematoxylin & eosin staining (HE) and immunofluorescence (IF) staining. The remaining mice (*n* = 4 per group) were anesthetized with an intraperitoneal injection of pentobarbital sodium, and skin samples from the original skin wound defects were obtained and used for molecular analyses. Then, the mice were sacrificed. The skin samples were rinsed with PBS and stored at −80 °C until use.

All experiments involving animals were conducted in a blinded manner.

### Histological analysis

The skin samples from the mice (*n* = 3 per group) perfused with 4% paraformaldehyde were rinsed with PBS, fixed with 4% paraformaldehyde, and embedded in paraffin, and paraffin sections (6 μm) were used for HE staining. Representative images were captured to visualize the pathological alterations, such as epidermal thickness, dermal microstructure, and skin appendages, in the area of skin regeneration. Photographs were captured at 10× and 20× magnification using a fully automatic fluorescence inverted microscope (Dmi8+DFC 7000 T, Leica, Germany).

### Western blot analysis

Skin samples from mice (*n* = 4 per group) were used for Western blot analysis. Levels of the Notch1, Notch2, Notch3, Jagged1, Jagged2, Hes1, HMGB1, and TNF-α proteins in the skin samples were analyzed using Western blotting as described in our previous study [25]. Briefly, skin samples from the experimental groups were lysed with RIPA buffer on ice and centrifuged at 13000 g for 20 min at 4 °C to extract total proteins. Then, 20 μg of total proteins were resolved on SDS–PAGE gels and transferred to PVDF membranes, followed by an overnight incubation at 4 °C with 4 ml of the appropriate dilutions of the following primary rabbit monoclonal antibodies: anti-Notch1 (1:1000; NB100-78486, Novus), anti-Notch2 (1:1000; 5732 S, Cell Signaling Technology), anti-Notch3 (1:1000; Ab23426, Abcam), anti-Jagged1 (1:1000; PA5-72843, Invitrogen), anti-Jagged2 (1:1000; 15–447, ProSci), anti-Hes1 (1:1000; sc-166378, Santa Cruz), anti-HMGB1 (1:1000; sc-74085, Santa Cruz); anti-TNF-α (1:1000; sc-52746, Santa Cruz), and anti-GAPDH (1:1000; ab8245, Abcam) as an internal control. Then, the membranes were washed three times and incubated at room temperature for 2 h with an HRP-conjugated goat anti-rabbit (074-1506, KPL) or goat anti-mouse (074-1806, KPL) antibody at a dilution of 1:10000. After the membranes were washed three times, the blots were visualized with ECL.

### IF staining

Skin samples from the mice (*n* = 3 per group) perfused with paraformaldehyde were postfixed in the same fixative for 2–4 h, transferred to 30% sucrose and incubated at 4 °C for 48 h, rinsed with PBS, embedded in O.C.T. compound (SAKURA.4583, SAKURA, United States), and sliced into frozen sections. Then, frozen sections (6 μm) were used for IF staining. The expression levels of Notch1, Notch2, Notch3, Jagged1, Jagged2, Hes1, CD31, α-smooth muscle actin (α-SMA), collagen I, and cytokeratin 14 were analyzed using IF staining. Two pairs of antibodies were used for double IF staining: CD31 and α-SMA, and collagen I and cytokeratin 14. Skin samples underwent gradient sucrose dehydration and embedding in O.C.T. compound. Then, the samples were incubated with the following anti-mouse primary antibodies, mouse anti-Notch1 (1:100; NB100-78486, Novus), rabbit anti-Notch2 (1:1000; 5732 S, Cell Signaling Technology); rabbit anti-Notch3 (1:200; Ab23426, Abcam), rabbit anti-Jagged1 (1:100; PA5-72843, Invitrogen), rabbit anti-Jagged2 (1:200; 15–447, ProSci), rabbit anti-Hes1 (1:200; sc-166378, Santa Cruz), mouse anti-CD31 (1:500; ab24590, Abcam), rabbit anti-α-SMA (1:100; ab5694, Abcam), rabbit anti-collagen I (1:400; ab34710, Abcam), or mouse anti-cytokeratin 14 (1:400; ab7800, Abcam) at 4 °C overnight, followed by rinses with PBS and an incubation with Cy3-conjugated secondary antibodies (donkey anti-rabbit, 1:400; 711-165-152, Jackson ImmunoResearch, West Grove, PA, United States) or FITC-conjugated secondary antibodies (donkey anti-mouse, 1:200; 715-095-150, Jackson ImmunoResearch, West Grove, PA, United States) for 1 h at room temperature. The sections were then examined with a confocal microscope (Leica TCS SP8, Germany).

### Statistical analysis

The data were analyzed using one-way analysis of variance followed by Tukey’s post-hoc test with SPSS 18.0 (SPSS Inc., Chicago, IL). The Shapiro–Wilk test was first used to check the normality of data. Data were considered normally distributed if *p* > 0.05. All data are presented as the means ± SEM. Differences were considered statistically significant at *p* < 0.05.

### Data available on request from the authors

The data that support the findings of this study are available from the corresponding author upon reasonable request.

## Results

### Formation and identification of spheroids composed of ADSCs

**Fig. 1:**
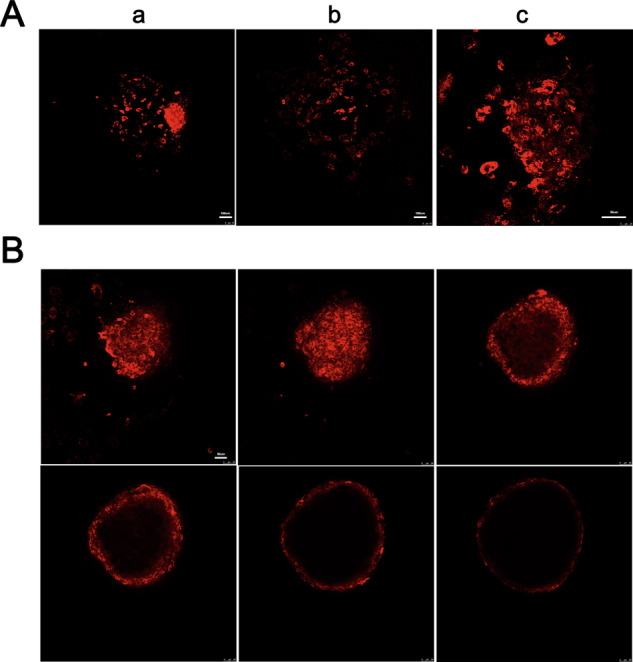
Assessment of the formation of spheroids composed of ADSCs and cell viability. **A** IF staining showed that the spheroids composed of ADSCs had good morphology and the cells migrated from the spheroid, suggesting that the ADSCs maintained good proliferation and migration abilities after spheroid formation. **B** Images of different cross-sections of the spheroids revealed that the cells were evenly distributed.

ADSCs were stained with CM-Dil and then cultured in ultralow attachment 96-well plates to confirm spheroid formation and cell viability. As shown in Fig. 1A, the spheroids composed of ADSCs formed after 2 days with good morphology, and the cells migrated from spheroids when cultured on the 2D dish, indicating that the cells maintained excellent proliferation and migration abilities. Furthermore, we observed an even distribution of ADSCs in the spheroids (Fig. 1B).

**Fig. 2:**
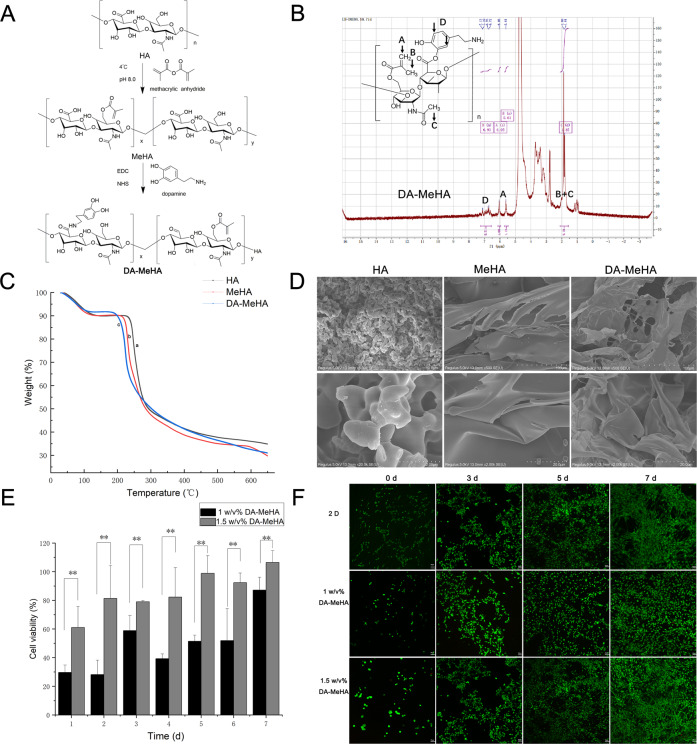
DA-MeHA synthesis, characteristics and biocompatibility. **A** The DA-MeHA synthesis process. **B** ^1^H NMR spectrum of DA-MeHA. **C** TGA thermograms of HA, MeHA, DA-MeHA and their blends with a temperature increase rate of 20 °C/min. (a) gray line—HA, (b) red line—MeHA, and (c) blue line—DA-MeHA. **D** SEM images of HA, MeHA and DA-MeHA. HA formed crystal-like structures while MeHA and DA-MeHA formed similar porous leaf-like structures. **E**, **F** CCK-8 assays and live/dead staining showed that the DA-MeHA hydrogel provides a biocompatible 3D culture environment for encapsulating ADSCs in vitro (*p < 0.05 and **p < 0.01).

### DA-MeHA synthesis, characteristics and biocompatibility

Given the low survival rates of ADSCs when applied alone [12], the DA-MeHA hydrogel biomaterial was designed to increase the survival and viability of ADSCs. HA was crosslinked with methacrylic anhydride to synthesize MeHA, and the product was crosslinked with dopamine hydrochloride to form DA-MeHA (Fig. 2A).

In the ^1^H NMR spectra of DA-MeHA, the prominent methyl peaks on the molecular chains of HA and methacrylic acid appeared at 1.81 ppm and 1.88 ppm; the double bond peaks introduced by the modification of methacrylic anhydride appeared at 5.61 ppm and 6.05 ppm; and the benzene ring peak introduced by the modification of dopamine appeared at 6.72 ppm, 7.10 ppm, and 7.13 ppm. The synthesized DA-MeHA had a methacrylic anhydride crosslinking degree of 50.0% and a dopamine crosslinking degree of 5.6% based on the calculated characteristic peak area (Fig. 2B). Light-activated crosslinking controls the degree of polymerization, and sequential polymerization allows the implantation of cells onto or into the DA-MeHA hydrogel.

### TGA of HA, MeHA and DA-MeHA

Changes in the thermal stabilities of the pure HA, MeHA, and DA-MeHA films were examined by TGA under nitrogen. The TGA curves for HA, MeHA, and DA-MeHA were similar in shape and each contained two intervals of rapid weight loss, as shown in Fig. 2C. For HA and MeHA, the first decrease in weight accounted for ~10.8% and was in the range of 30–125 °C due to the loss of moisture and residual acetic acid. In comparison, the first DA-MeHA weight change occurred at 30–118 °C and was ~8.1%. HA, MeHA and DA-MeHA mainly decomposed in the ranges of 225–300 °C, 200–300 °C, and 176 °C–300 °C showing weight losses of ~41%, 42 and 42%, respectively.

### The morphologies of HA, MeHA and DA-MeHA

The SEM images revealed crystal-like structures of HA, while MeHA and DA-MeHA exhibited similar porous leaf-like structures (Fig. 2D). Apparently, this change in the structure of modified HA was attributed to crosslinking with methacrylic anhydride. After the introduction of dopamine, no essential difference was observed between the morphologies of MeHA and DA-MeHA.

### In vitro assessment of DA-MeHA biocompatibility

The results of the CCK-8 assay showed that the cell density of the 1.5 w/v% DA-MeHA group was close to that of the 2D group on the 5th day; on the 7th day, the cell density was higher than that of the 2D group and significantly higher than that of the 1.0 w/v% DA-MeHA group (**p < 0.01). The DA-MeHA hydrogel exhibited efficient biocompatibility and low biological toxicity, and we found that the best concentration ratio of the DA-MeHA hydrogel was 1.5 w/v% (Fig. 2E).

Live/dead staining assays showed that the cell density of the 1.5 w/v% DA-MeHA group was similar to that of the 2D group on the 3^rd^ day and higher than that of the 2D group on the 7th day. The cells may have passed through the 1.5 w/v% DA-MeHA hydrogel to form a 3D culture model, which was helpful for increasing the cell density (Fig. 2F). Thus, the DA-MeHA hydrogel had low cytotoxicity and good biocompatibility and was suitable for the culture of ADSCs.

### In vivo experiment

#### The DA-MeHA hydrogel can be used as an effective stem cell carrier

**Fig. 3:**
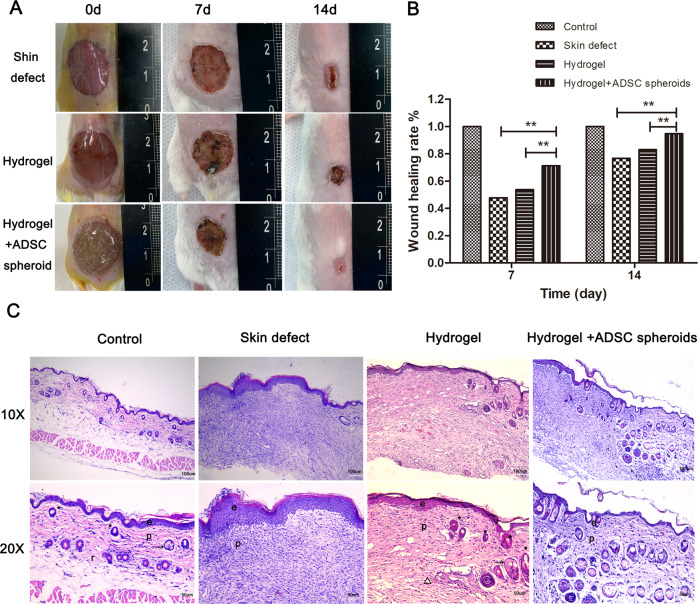
The DA-MeHA hydrogel encapsulating spheroids composed of ADSCs accelerated the regeneration of skin defects in mice. **A** Images captured on the 7th and 14th days after surgery showed that the skin defect healed remarkably faster after treatment with DA-MeHA hydrogel-encapsulated spheroids composed of ADSCs than the other groups. **B** Wound healing rates. The wound healing rate of the hydrogel+ADSCs spheroid group was significantly higher than that of the skin defect group and hydrogel group. **C** HE staining showed that the structure and morphology of the regenerated epidermis and dermis in the hydrogel + ADSCs spheroid group were closest to those of the control group, followed by the hydrogel group. Abbreviations: e epidermis, p papillary dermis, and r reticular dermis. Arrowheads indicate sebaceous glands and asterisks indicate hair bulbs (*p < 0.05 and **p < 0.01).

Having confirmed the good biocompatibility between DA-MeHA and ADSCs, determining the effects of the ADSCs embedded in the DA-MeHA hydrogel on wound repair was urgently needed. Data from 1 mouse in the hydrogel group was missing because it did not survive surgery. Interestingly, the in vivo experiments showed that the wound dressed with the DA-MeHA hydrogel encapsulating ADSCs healed faster than the skin defect group and hydrogel group, which is consistent with the wound closure rate (Fig. 3), indicating that the DA-MeHA hydrogel encapsulating ADSCs had a positive effect on skin regeneration.

**Fig. 4:**
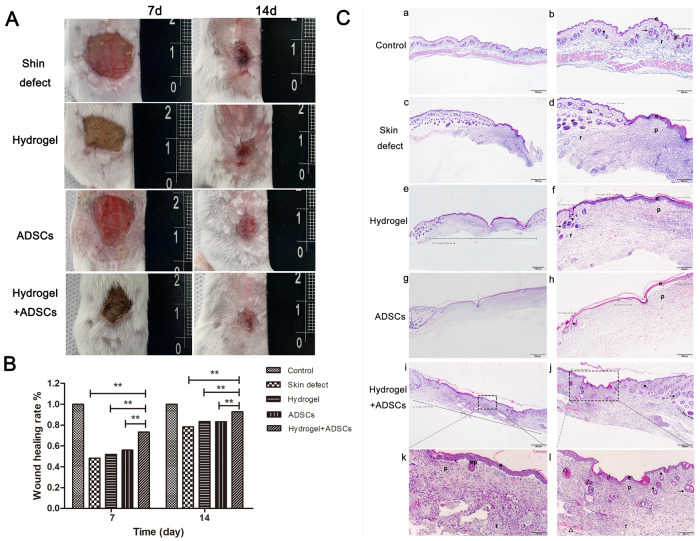
DA-MeHA hydrogels encapsulating suspended ADSCs accelerated skin regeneration in mice better than injection of ADSCs alone. **A** Images captured on the 7th and 14th days after surgery showed that wounds treated with DA-MeHA hydrogel-encapsulated suspensions of ADSCs healed remarkably faster than those in the other groups. **B** Wound healing rates. The wound healing rate in the hydrogel+ADSCs group was significantly higher than that in the skin defect, hydrogel, and ADSCs groups. **C** HE staining showed that the regenerated epidermal and dermal structures and their morphologies in the hydrogel + ADSCs group were closest to those of the control group. The epidermal papillary layer formed gradually in the center of the wound in only the hydrogel+ADSCs group (C-k), and newborn intradermal hair follicles and enhanced neovascularization were gradually observed (C-l). Abbreviations: e epidermis, p papillary dermis, and r reticular dermis. Arrowheads indicate sebaceous glands, asterisks indicate hair bulbs, and triangles indicate areas of neovascularization (*p < 0.05 and **p < 0.01).

Another animal experiment was conducted to further clarify the effect of either spheroids composed of ADSCs or DA-MeHA hydrogels on the promotion of skin regeneration. The results from 1 mouse in the skin defect group were missing because it did not survive 1 d after surgery. As presented in Fig. 4, on the 7th day, a significant increase in the wound healing rate was observed in the hydrogel+ADSCs group compared to the ADSCs group. On the 14th day, both the hydrogel+ADSCs and ADSCs exerted positive effects on wound closure, while the wound closure rate of the hydrogel+ADSCs group was significantly higher than that of the ADSCs group (Fig. 4). Our results suggested that the ADSCs encapsulated in the DA-MeHA hydrogel accelerated skin regeneration better than injection of ADSCs alone. Furthermore, HE staining revealed that only the sample from the hydrogel+ADSCs group formed an epidermal papillary layer in the center of the skin defect area compared with the other groups (Fig. 4C–k). Additionally, more intradermal hair follicles and new blood vessels formed (Fig. 4C–l) in the hydrogel+ADSCs group than in the other experimental groups.

**Fig. 5:**
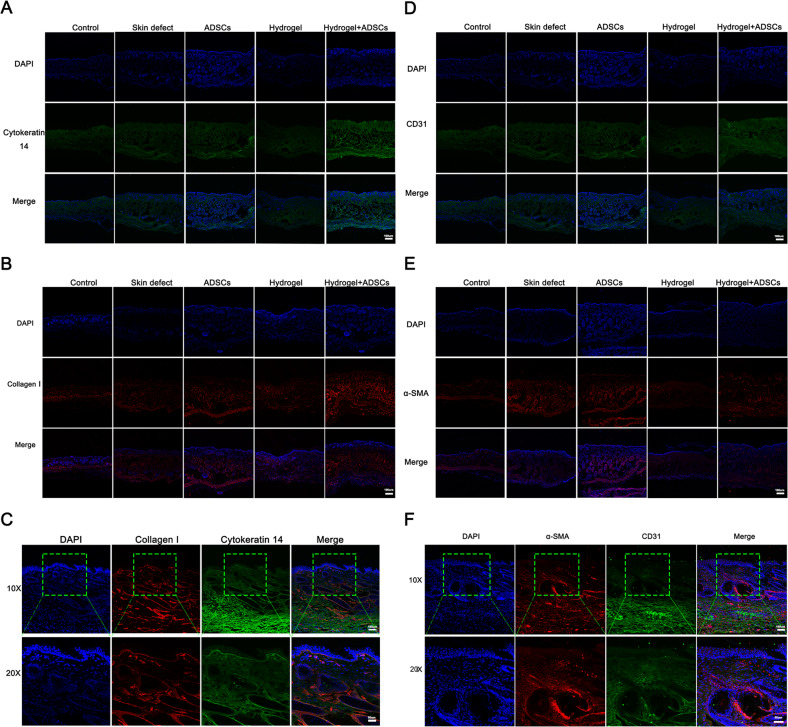
DA-MeHA hydrogel-encapsulated ADSCs promoted epidermal regeneration and dermal collagen formation and accelerated neovascularization. **A** Cytokeratin 14 (CK14) is a surface marker of the epidermis the is used to evaluate skin defect re-epithelialization. IF staining showed higher CK14 expression in the hydrogel+ADSCs group than in the other groups. **B** Considering the migration of fibroblasts to the wound area, the collagen I level was measured to evaluate the therapeutic effects of the different treatments consisting of the DA-MeHA hydrogel and ADSCs. IF staining showed higher collagen I expression in the hydrogel+ADSCs group than in the other groups. **C** Images of IF staining from the hydrogel + ADSCs group showed that CK14 and collagen I were coexpressed in the skin. **D** CD31 is a surface marker of vascular endothelial cells. IF staining showed higher CD31 expression in the hydrogel+ADSCs group than in the other groups. **E** α-SMA is a specific marker of myofibroblasts (MFB) that is used to evaluate skin defect re-epithelialization. IF staining revealed higher α-SMA expression in the hydrogel+ADSCs group than in the other groups. **F** IF staining of the hydrogel + ADSCs group showed that α-SMA and CD31 were coexpressed in the regenerated skin, indicating that the ADSCs embedded in the DA-MeHA hydrogel retained a good ability to promote angiogenesis, and the effect was better than that of injection of ADSCs.

Two different animal experiments were conducted as described above, and the results showed that the DA-MeHA hydrogel maintained the functions of suspensions or spheroids composed of ADSCs to promote skin wound regeneration and skin remodeling compared with injection of ADSCs. IF staining also showed that DA-MeHA hydrogel-encapsulated ADSCs promoted epidermal regeneration and dermal collagen formation (Fig. 5A–C), simultaneously accelerating microvascular endothelial cell activity and neovascularization (Fig. 5D–F). Thus, the DA-MeHA hydrogel used to carry ADSCs might enhance the interaction of dermal fibroblasts and endothelial cells, thereby increasing angiogenic activity within the skin wound.

#### The DA-MeHA hydrogel inhibits the inflammatory response, which is potentially modulated by Notch signaling

**Fig. 6:**
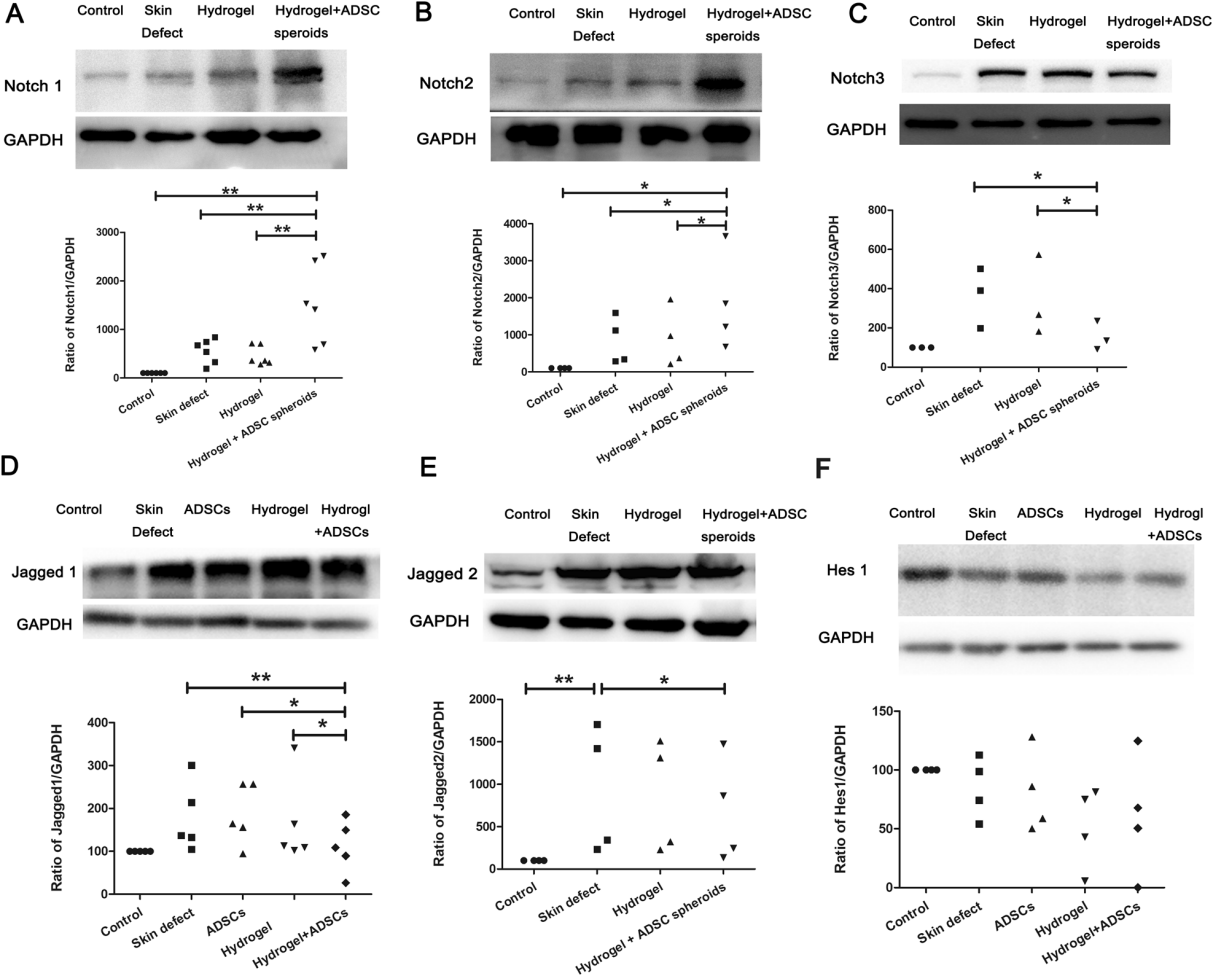
The DA-MeHA hydrogel regulated Notch signaling. **A**–**E** DA-MeHA hydrogel-encapsulated spheroids or suspensions of ADSCs upregulated the expression of Notch1 and Notch2 while downregulating the expression of Notch3, Jagged1 and Jagged2. **F** No significant difference in Hes1 expression was observed among the groups. *n* = 4 samples/group (*p < 0.05 and **p < 0.01).

Based on the aforementioned data, the DA-MeHA hydrogel maintained the paracrine functions of spheroids or suspensions composed of ADSCs and enhanced the regenerative repair ability of local skin stem cells around the skin wound defect, which may involve Notch signaling. Spheroids or suspensions of ADSCs embedded in DA-MeHA hydrogels upregulated the expression of Notch1 and Notch2 while downregulated the expression of Notch3, Jagged1 and Jagged2 (Fig. 6A–E**)**. A significant difference in Hes1 expression was not observed among the groups (Fig. 6F). The Notch signaling pathway, which helps regulate the migration, proliferation and differentiation of cells [28, 29], modulates the adhesive ability of keratinocytes and controls skin cell differentiation [29]. We speculated that local stem cells in skin receive cues from their niche and from transplanted ADSCs to enhance wound repair by regulating Notch signaling [30].

**Fig. 7:**
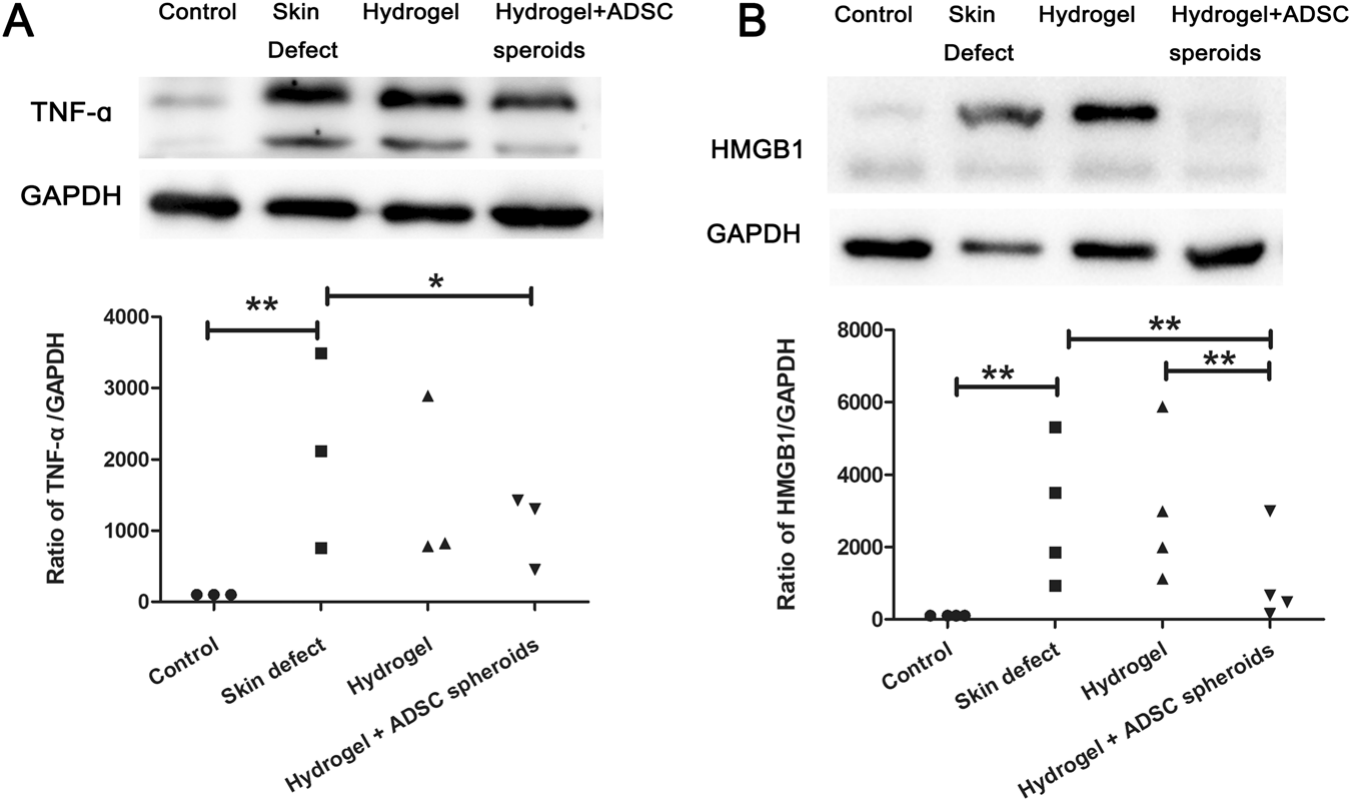
DA-MeHA hydrogel-encapsulated spheroids composed of ADSCs downregulated inflammatory responses. **A** DA-MeHA hydrogel-loaded spheroids composed of ADSCs downregulated the expression of TNF-α. **B** DA-MeHA hydrogel-loaded spheroids composed of ADSCs downregulated the expression of HMGB1. *n* = 4 samples/group (*p < 0.05 and **p < 0.01).

Epithelial stem cells (EpSCs) are vulnerable to inflammatory pressures [30], and spheroids composed of ADSCs encapsulated by DA-MeHA hydrogels downregulated the expression of inflammatory factors (TNF-α and HMGB1), suggesting that the DA-MeHA hydrogels effectively inhibited inflammation (Fig. 7).

## Discussion

Skin regeneration is one of the most complex processes in the human body [6]. Notably, the local wound environment is primarily considered the key target for therapeutic strategies. Alterations in the wound microenvironment, including mechanical forces, ECM components, growth factors and oxygen contents, directly modulate the recruitment and activation of stem cells and subsequently alter skin regeneration [6]. Diabetes, infections and other pathological conditions reduce the recruitment of circulating stem cells [6]. Exogenous stem cells play a particularly important role in chronic wounds.

ADSCs are known to secrete endogenous factors such as vascular endothelial growth factor and prostaglandin E2 that promote angiogenesis, modulate the inflammatory microenvironment, and initiate the wound repair process [12, 31–33]. However, low cell survival under substantial pathological conditions, such as large skin defects, is the main reason for the low efficacy of stem cell therapy [8, 12]. Spheroids composed of MSCs were shown to spontaneously form in ultralow adsorption 96-well plates and therefore maintain cell–cell junctions via self-assembly, similar to the clustering process of stem cells in vivo [34], enabling them to adapt to hypoxic conditions or to wounds with a poor blood supply [35, 36]. Stem cell spheroids exhibit greater secretion of trophic factors than suspended cells [7, 36]. In the present study, spheroids composed of ADSCs formed (Fig. 1) and were applied to treat full-thickness skin defect wounds. The maintenance of these spheroids on wounds is challenging, and a biomaterial that can be used as a carrier for stem cell spheroid transplantation is urgently needed.

With suitable structures and swelling ratios, HA hydrogels are highly advantageous, as they provide oxygen, absorb exudates and maintain a moist healing environment [20]. More importantly, HA is an essential component of the ECM and regulates cell proliferation, motility, and collagen composites [6]. We covalently crosslinked HA with a methacrylate group to obtain a curable and slowly metabolized hydrogel (MeHA) that would overcome the inherent disadvantages of HA, such as poor mechanical properties and low resistance to enzymatic degradation [19]. However, the MeHA hydrogel does not meet the requirement that the hydrogel effectively adheres to the skin wound and is not easily peeled off. Dopamine is a bioadhesive molecule with strong adhesion to various substances even in aqueous solutions [37]. Hence, in our study, MeHA was conjugated to dopamine to obtain an adhesive hydrogel (DA-MeHA), and its curability, injectability, porous structure, and biocompatibility were characterized (Fig. 2). The DA-MeHA hydrogel firmly adhered to the wound and cured defects in situ. The TGA results showed that the onset of rapid weight loss occurred sooner after administration of DA-MeHA than HA and MeHA, but the weight loss rates during the heating process were similar in all three samples.

Our in vitro and in vivo experiments revealed the biocompatibility and biological activity of the DA-MeHA hydrogel. The in vitro experiments suggested that the ADSCs embedded in the DA-MeHA hydrogel maintained a good proliferation ability, and the cell survival rate was higher than that of cells cultured in a 2D environment **(**Fig. 2E, F**)**. Specifically, the in vivo experiments showed that the DA-MeHA hydrogel loaded with suspensions of ADSCs accelerated wound closure and performed significantly better than ADSCs injected into the wound edge (Fig. 4), potentially providing a new strategy for the therapeutic delivery of stem cells.

During the remodeling process, the dermal fibroblasts deposit new ECM proteins, such as collagen I, to strengthen the repaired tissue [38]. In our study, IF staining showed that the DA-MeHA hydrogel encapsulating ADSCs promoted epidermal regeneration and dermal collagen formation **(**Fig. 5A–C**)**, simultaneously accelerating neovascularization (Fig. 5D–F). Resident skin stem cells are programmed into an active state by their niches and the factors secreted from ADSCs to accelerate wound healing [30]. Regarding spheroids or suspensions of ADSCs encapsulated in DA-MeHA hydrogels, paracrine mechanisms might play a major role in triggering wound repair. HE staining revealed remodeling of the epidermal and dermal microstructures of the regenerated skin in the hydrogel+ADSCs group **(**Fig. 4C**)**. Notably, the epidermal papillary layer formed gradually in the center of the wound (Fig. 4C–k), and newborn intradermal hair follicles and increased neovascularization were gradually observed in the center of the wound in only the hydrogel+ADSCs group (Fig. 4C–l). Initial dermal regeneration relies on cells of the reticular dermis and hypodermis, which is characterized by ECM with collagen fibers that do not induce hair follicle regeneration [39]. The upper dermal lineage forms only the papillary dermis until re-epithelialization, which illustrates the deficiency of hair follicles in freshly closed wounds [39]. The emergence of regenerated hair follicles and dermal papilla in the center of the wound area (Fig. 4C–l) may suggest that ADSCs embedded in the DA-MeHA hydrogel significantly accelerated skin remodeling [16]. ADSCs encapsulated by the DA-MeHA hydrogel may differentiate into primary mesenchymal cells, accelerate the regeneration of surrounding hair follicles, and simultaneously induce new blood vessel generation. The basal cells of the newborn squamous epithelium subsequently form dermal papillae, which in turn increase hair follicle formation.

The proliferation and differentiation of skin stem cells mainly depend on their niches, which mainly include cells, blood vessels, the ECM, three-dimensional space and signaling pathways [40, 41]. Among these signaling pathways, Notch signaling plays a crucial role in regulating and maintaining skin homeostasis [40]. Notch signaling is essential for spinous and granular cell differentiation, maintaining hair follicle homeostasis and accelerating skin barrier formation [42, 43]. In the mouse epidermis, the receptors Notch1, Notch2, Notch3 and ligand Jagged1 are expressed in the suprabasal layer, and the ligand Jagged2 is expressed in the basal layer [29]. The downregulation of Notch signaling proteins impairs epidermal reformation, collagen arrangement and skin appendage regeneration [28]. The Notch1-Jagged1 pathway also plays a role in promoting hair follicle stem cells (HFSCs) function [44]. Notch signaling controls the migration of cells from the basal proliferative layer into the spinous layer in the epidermis [44]. In the present study, spheroids or suspensions of ADSCs embedded in DA-MeHA hydrogels upregulated the expression of Notch1 and Notch2 and downregulated the expression of Notch3, Jagged1 and Jagged2 (Fig. 6A–E) compared with the skin defect group and ADSCs group. No significant difference in Hes1 expression was observed among the groups (Fig. 6F). We speculate that local stem cells in skin receive cues from their niche and from transplanted ADSCs to enhance wound repair by regulating Notch signaling [30]. Notch signaling resembles an intricate interwoven network rather than a simple linear pathway [45]. Our results may indicate that Notch signaling increases the differentiation of skin stem cells via a Hes1-independent mechanism. High Notch signaling levels promote the differentiation of epidermal stem cells (EpSCs) into interfollicular lineages [28]. Activation of Notch2 and Notch3 stimulates terminal differentiation, and Jagged1 and Jagged2 also function as terminal differentiation–inducing stimuli [46]. Hence, we hypothesized that the upregulated expression of Notch1 in the hydrogel+ADSCs group contributes to basal cell proliferation and further accelerates epidermal regeneration. In addition, Notch2 and Notch3 cooperate to induce the terminal differentiation of interfollicular epidermal cells.

The immune profile of ADSCs and their potential shift toward an anti-inflammatory phenotype are critical for the proliferation and remodeling stages of healing [11]. In addition to the effects of terminal skin cell differentiation, Notch signaling also controls the inflammatory response in the skin [43]. TNF-α alters the physiological function of epidermal cells and dermal fibroblasts to ultimately slow re-epithelialization and wound closure [15]. Loss of Notch activity, including Notch1 and Notch2, leads to the release of proinflammatory cytokines, resulting in chronic inflammation [43]. We found that the DA-MeHA hydrogel or DA-MeHA hydrogel encapsulating ADSCs significantly decreased the expression of the proinflammatory cytokines HMGB1 and TNF-α in skin defect wounds. Therefore, we hypothesized that the DA-MeHA hydrogel abolished the inflammatory response of the microenvironment to promote the survival of both transplanted and endogenous cells, which may be related to Notch signaling.

In conclusion, we developed a biocompatible material (DA-MeHA hydrogel). The ADSCs embedded in the DA-MeHA hydrogel had sufficient paracrine function and the DA-MeHA hydrogel enhanced, modulated or even initiated skin repair processes mediated by ADSCs. These data suggest that DA-MeHA hydrogels will become therapeutic stem cell carriers in clinical practice and will be widely applied in various stem cell therapies.

In this study, mice were used as an animal model to study the effect of the DA-MeHA hydrogel as a stem cell carrier on skin regeneration and repair, but the data may be biased due to the possible species differences between mice and humans. However, animal models exclusively provide definitive evidence for the animal being investigated and provide evidence for processes occurring in humans. Animal models are very beneficial for studying the mechanisms underlying human conditions [47]. Various animal models and even clinical trials are required in the future to enhance the valuable translational potential and application of DA-MeHA hydrogels in clinical practice.

## Conclusions

The DA-MeHA hydrogel developed here is a potential candidate carrier for stem cell therapy. The DA-MeHA hydrogel was nontoxic and biocompatible and had good mechanical properties and viscoelasticity. Loading stem cells into the DA-MeHA hydrogel stimulated early angiogenesis in wounds and promoted wound healing and skin remodeling in vivo. This hydrogel represents a noninvasive, economical and efficient wound management tool based on stem cell therapy. However, this study failed to clearly elucidate the mechanism by which the DA-MeHA hydrogel enhances the function of ADSCs. Further study is needed to clarify the underlying mechanism.

## Supplementary information

WB-HES1

WB-HMGB1

WB-Jagged1

WB-Jagged2

WB-Notch1

WB-Notch2

WB-Notch3

WB-TNF-a

AJ-ckecklist
