# Supplementary figures and images for: A dopamine-methacrylated hyaluronic acid hydrogel as an effective carrier for stem cells in skin regeneration therapy
Source: Cell Death Dis. 2022 Aug 27;13(8):738. doi: 10.1038/s41419-022-05060-9 (PMC9420120; doi:10.1038/s41419-022-05060-9)

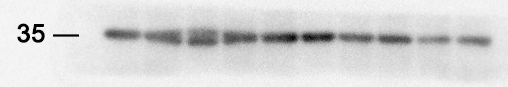

Supplement: Supplementary file 1 — WB-HES1 [file 41419_2022_5060_MOESM1_ESM.tif]

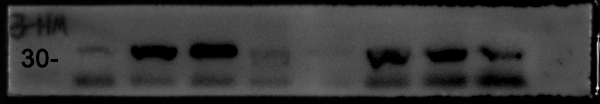

Supplement: Supplementary file 2 — WB-HMGB1 [file 41419_2022_5060_MOESM2_ESM.tif]

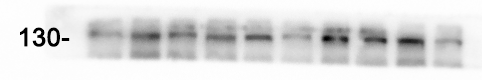

Supplement: Supplementary file 3 — WB-Jagged1 [file 41419_2022_5060_MOESM3_ESM.tif]

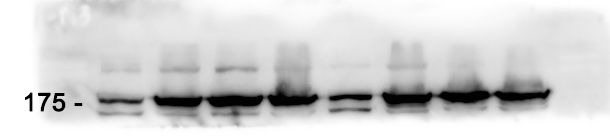

Supplement: Supplementary file 4 — WB-Jagged2 [file 41419_2022_5060_MOESM4_ESM.tif]

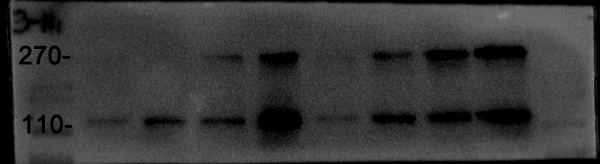

Supplement: Supplementary file 5 — WB-Notch1 [file 41419_2022_5060_MOESM5_ESM.tif]

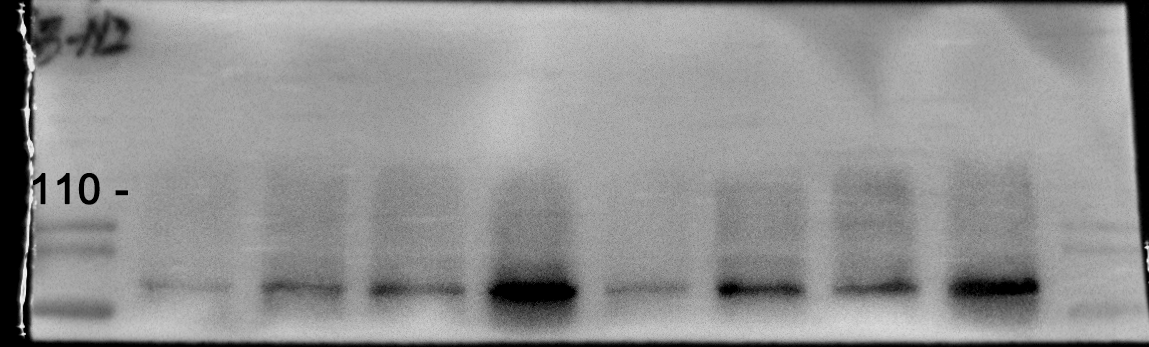

Supplement: Supplementary file 6 — WB-Notch2 [file 41419_2022_5060_MOESM6_ESM.tif]

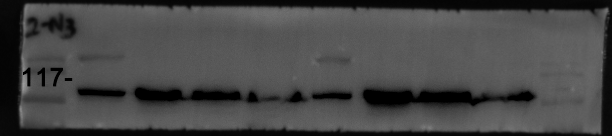

Supplement: Supplementary file 7 — WB-Notch3 [file 41419_2022_5060_MOESM7_ESM.tif]

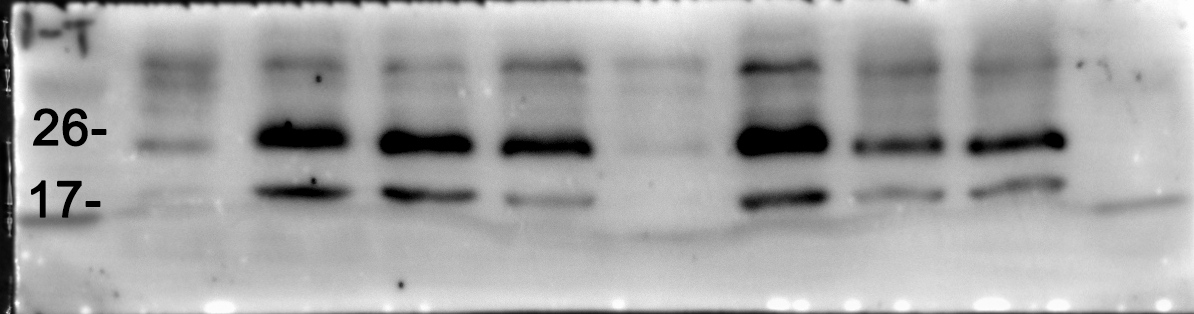

Supplement: Supplementary file 8 — WB-TNF-a [file 41419_2022_5060_MOESM8_ESM.tif]
